# Supplementary material for: Psychosocial Aspects of Living Long Term with Advanced Cancer and Ongoing Systemic Treatment: A Scoping Review
Source: Cancers (Basel). 2022 Aug 11;14(16):3889. doi: 10.3390/cancers14163889 (PMC9405683; doi:10.3390/cancers14163889)
Supplement: Supplementary file 1 [file cancers-14-03889-s001.zip › cancers-1825415-supplementary/Supplementary File S1. Search strategy.pdf]

Table S1. Search strategy (Medline).

	**Patients**
1	((cancer OR cancers OR oncolog* OR neoplasm* OR tumor OR tumors OR tumour OR tumours OR malignan* OR melanoma OR melanomas OR sarcoma OR sarcomas OR carcinoma* OR GIST) ADJ3 (advance* OR metasta* OR dissemin* OR progress* OR incurab* OR inoperab* OR chronic OR recurr* OR relaps* OR palliative* OR protracted)).ti,ab,kf.
2	exp neoplasm metastasis/
3	gastrointestinal stromal tumors/
	**Treatment**
4	((ongoing OR continu* OR long-term OR long term) ADJ3 (treatment OR treatments OR medicine OR medicines OR medication OR therapy OR therapies OR drug or drugs OR agent OR agents)).ti,ab,kf.
5	"molecular targeted therapy"/ OR "precision medicine"/
6	((molecular OR targeted OR individuali* OR personali* OR selective OR precision) ADJ3 (treatment OR treatments OR medicine OR medicines OR medication OR therapy OR therapies OR drug OR drugs OR agent OR agents)).ti,ab,kf.
7	exp immunotherapy/
8	(immunotherapy OR immuno-therapy OR immunotherapies OR immuno-therapies OR Immunosuppress* OR immuno-suppress*).ti,ab,kf.
9	((antibody OR antibodies) ADJ3 (therapy OR therapies OR treatment OR treatments OR drug OR drugs OR monoclonal OR immunomodulatory)).ti,ab,kf.
10	(systemic ADJ3 (treatment OR treatments OR medicine OR medicines OR medication OR therapy OR therapies OR drug OR drugs OR agent OR agents)).ti,ab,kf.
11	exp hormone antagonists/
12	((hormon* OR antihormon* OR anti-hormon* OR endocrine) ADJ3 (therapy OR therapies OR treatment OR treatments OR drug OR drugs OR antagonist OR antagonists OR agonist OR agonists OR suppression)).ti,ab,kf OR ((androgen OR estrogen OR oestrogen) ADJ3 (antagonist OR antagonists OR "deprivation therapy")).ti,ab,kf.
13	exp therapies, investigational/
14	((experimental OR investigational OR innovative OR novel) ADJ3 (treatment OR treatments OR medicine OR medicines OR medication OR therapy OR therapies OR drug OR drugs OR agent OR agents)).ti,ab,kf.
15	("tyrosine kinase inhibitor" OR "tyrosine kinase inhibitors" OR "tyrosine-kinase inhibitor" OR "tyrosine-kinase inhibitors" OR TKI OR TKIs).ti,ab,kf.
	**Psychosocial aspects**
16	"mental health"/ OR exp "mental disorders"/ OR patients/px [Psychology]
17	(psycho* OR psychiat* OR "mental disorder*").ti,ab,kf.
18	exp Stress, Psychological/
19	distress*.ti,ab,kf.
20	exp Emotions/
21	(mood OR emotion* OR Depression OR depressiv* OR fear OR fears OR anxi* OR worry OR worries OR bipolar OR "posttraumatic stress" OR "post-traumatic stress" OR PTSD).ti,ab,kf.
22	exp adaptation, psychological/

23	coping.ti,ab,kf OR resilience.ti,ab,kf.
24	*"quality of life"/
25	("quality of life" OR QOL OR HR-QOL OR HRQL OR HR-QL OR HRQOL).ti,ab,kf.
26	"social behavior"/ OR exp "social support"/
27	((social) ADJ3 (support OR 2ehaviour OR behaviors OR behaviour OR behaviours OR adjustment OR competence OR competences OR interaction OR interactions OR participation OR connection OR connections OR relationship OR relationships OR network OR networks OR environment OR environments)).ti,ab,kf.
28	uncertainty/
29	(uncertainty OR uncertainties).ti,ab,kf.
30	hope/
31	hope*.ti,kf OR (patient* ADJ hope*).ab.
32	trust/
33	trust.ti,kf.
34	optimism/
35	optimism.ti,ab,kf.
36	exp grief/
37	grief.ti,ab,kf.
38	(expectation* OR expectanc*).ti,kf OR (patient* ADJ3 (expectation* or expectanc*)).ab
39	(patient* ADJ experience*).ti,kf.
40	needs.ti,kf OR ((unmet OR support OR care OR healthcare OR information OR support OR patient*) ADJ needs).ab
41	concern*.ti,kf. OR (patient* ADJ concerns).ab
