# Supplementary material for: Psychosocial Aspects of Living Long Term with Advanced Cancer and Ongoing Systemic Treatment: A Scoping Review
Source: Cancers (Basel). 2022 Aug 11;14(16):3889. doi: 10.3390/cancers14163889 (PMC9405683; doi:10.3390/cancers14163889)
Supplement: Supplementary file 1 [file cancers-14-03889-s001.zip › cancers-1825415-supplementary/Supplementary File S3. Data-extraction tables.pdf]

Data-extraction tables

Table S2. Data-extraction table of quantitative studies.

Author	Study design	Psychosocial outcomes	Recruitment	Patients	Sample size	Mean age (years)	Female sex (%)	Time since diagnosis	Baseline results (mean (SD))	Terminology to refer to patient group
**Adamowicz, 2018, Poland**	Prospective cohort study	Health-related Quality of Life	Consecutive sampling; recruitment from the Specialist Hospital and the Regional Center of Oncology in Poland	Colorectal cancer; first-line palliative chemotherapy	330	66.0 (SD=11.7, range: 36-88)	57.9	NR	*EORTC-QLQ-C30* Pre-treatment:<br>QL: 65 (16.5)<br>EF: 71 (15.6)<br>Post-treatment:<br>QL: 68 (15.4)<br>EF: 72 (14.8)	Metastatic colorectal cancer patients; patients with advanced disease
**Bannink, 2008, The Netherlands**	Longitudinal survey study	Depression; anxiety	Part of the patients were recruited via the intervention arms of larger RCTs (EORTC 19881; EORTC 30012); recruitment of other patients not further specified	High-risk melanoma (n=8) or disseminated renal-cell carcinoma (n=35); interferon-alpha treatment	43	Median age: 58.0 (range: 36-72)	58.2	NR	*MADRS*<br>*Total* 3.9 (3.8)<br>*BAS Irritability*<br>0.4 (0.7)<br>*HADS-A*<br>4.6 (3.0)<br>*HADS-D*<br>2.6 (2.2)<br>*BDI sum score*<br>5.6 (4.1)<br>*SCL-90 Total*<br>112.6 (16.9)	Disseminated renal cell carcinoma
**Benedict, 2014, USA**	Cross-sectional preliminary analysis of baseline data from a larger randomized controlled trial	Psychosocial wellbeing; Quality of Life	Patients were part of a larger RCT; recruitment via referrals from urology clinics, community presentations, and through the Florida Cancer Data System	Prostate cancer; luteinizing hormone-releasing hormone agonists with concomitant external beam radiation therapy (EBRT)	80	69.7 (SD=9.6)	0.0	37.6 months (SD: 34.3)	*FACT-G*<br>*QL:* 82.7 (14.4)<br>*CES-D* 9.3 (9.5)	Patients with advanced prostate cancer undergoing androgen deprivation therapy
**Bergerot, 2019, USA**	Cross-sectional survey study	Quality of Life; anxiety; depression	Recruitment via one of three genitourinary medical oncologists at the institution	Metastatic renal cell carcinoma (63%), urothelial cancer (28%) and prostate cancer (8%); immunotherapy (checkpoint inhibitors (CPIs)): nivolumab (35%) and atezolizumab	60	65.0 (SD=13, rang: 31-91)	32.0	NR	*FACT-G PROMIS-Depression PROMIS-Anxiety* baseline scores NR	Patients with advanced genitourinary cancers initiating immunotherapy; patients with metastatic genitourinary cancer

				(30%), administered as first (33%) or second line of therapy (45%) for the majority of patients						
**Carmichael, 2013, USA**	Cross-sectional survey study	Health-related Quality of Life	Purposive sampling; all patients diagnosed with RCC were identified using a institutional database	Metastatic renal cell carcinoma; targeted therapy (VEGF- or mTOR-directed therapy)	28	58.0 (rang: 40-72)	250	NR	*EORTC-QLQ-C30* QL 73.5 (21.0)<br>EF 82.4 (14.6)	Long-term survivors (with metastatic renal cell carcinoma)
**Cohen, 2001, USA**	Secondary analysis of data from a phase Ib trial	Treatment-specific optimism; depression; mood disturbance; distress	Patients were enrolled in a phase Ib trial of a heat shock protein peptide complex 96 trial	Metastatic renal cell carcinoma (n=24), metastatic melanoma (n=22); nontoxic, active, specific immunotherapy comprised of an autologous tumor preparation without adjuvant therapy	46	54.9 (SD=8.9, range: 36-76)	30.0	Mean TSD: 23.2 months (SD: 37.1, range: 1-168)	*CES-D* 12.67 (9.78)<br>*POMS* TMD*:* 23.50 (33.71)<br>*POMS* Depression 8.33 (10.36)<br>*BSI* GSI 0.46 (0.40)<br>Depression 0.48 (0.68)	Patients with metastatic renal cell carcinoma/metastatic melanoma
**Cohen *(de Moor), 2002, USA***	Prospective observational study of data from a phase Ib trial	Quality of Life	Patients were enrolled in a phase Ib trial of a heat shock protein peptide complex 96 trial	Stage IV metastatic renal cell carcinoma; heat-shock protein peptide complex 96 (HSPPC-96) autologous tumor vaccine treatment, a nontoxic, active, specific immunotherapy	29	56.5 (SD=8.4, range: 37-76)	22.0	NR	*SF-36* GH: 62 (22);<br>Role limitations: emotional: 76 (38) *IES* 21 (17)	Patients with metastatic renal cell carcinoma
**Cohen *(Parker), 2002, USA***	Prospective observational study of data from a phase Ib trial	Quality of Life	Patients were enrolled in a phase Ib trial of a heat shock protein peptide complex 96 trial.	Advanced melanoma stage III (n=10) or stage IV (n=20); heat-shock protein peptide complex 96 (HSPPC-96) autologous tumor vaccine treatment, a nontoxic, active, specific immunotherapy	30	50.1 (SD=11.1)	37.0	NR	*SF-36* GH: 59 (21);<br>Role limitations: emotional: 91 (32) *IES* 23 (15)	Patients with advanced melanoma
**Custers, 2015, The Netherlands**	Cross-sectional survey study	Fear of cancer recurrence; fear of progression	Recruitment from the database in Radboud University Medical Centre	Gastrointestinal stromal tumors: localized (50%) or metastatic (50%); the tyrosine kinase inhibitor imatinib	54	Median age: 63.0 (range: 21-83)	46.0	Mean TSD: 3.9 years (range: 0.5-17)	*FCRI Severity* 16.8 (7.7)<br>*CWS* 14.46 (5.2)<br>*EORTC-QLQ-C30* QL 77.2 (19.8)<br>EF 78.4 (25.4)	Patients with metastatic GIST

									HADS 9.3 (7.8)<br>*IES* 15.1 (16.7)	
**Daugherty, 2005, USA**	Cross-sectional survey study	Spirituality; Quality of Life	Recruitment from the population of advanced cancer patients who had been accepted for phase I trials at a large urban, Midwestern medical center in the USA	Advanced cancer patients; experimental agents in clinical phase I trials (n=162) or standard care (n=156)	318	57.8 (SD=12.6) versus 57.9 (SD=12.3) for patients in phase I trials versus standard care respectively	45 versus 58	NR	*FACIT-SP* 40.7 (7.2) versus 39.7 (7.2)<br>*FACT-G*<br>QL 90.1 (13.6) versus 87.4 (14.5)<br>EW 15.6 (3.7) versus 16.2 (3.7) for patients in phase I trials versus standard care respectively	Advanced cancer patients; terminally ill cancer patients;
**Denouel, 2018, France**	Longitudinal survey study	Quality of Life	Patients were enrolled in the COG-ANGIO study in 3 French institutions	Metastatic renal cell carcinoma; antiangiogenic therapies (vascular endothelial growth factor or mTOR inhibitors)	39	Median age: 59.0 (28-78)	26.0	NR	*FACT-G*<br>QL 81.2<br>EW 18.2 (3.5) (11.0)	Metastatic renal cell carcinoma patients
**Devine, 2003, USA**	Longitudinal survey study	Quality of Life; psychological distress; social support; intrusive thoughts; avoidance	Patients were enrolled in a clinical trial receiving an experimental vaccine (Phase 1/b)	Metastatic renal cell carcinoma (n=29) and metastatic melanoma (n=24); non-toxic, active, specific immunotherapy, consisting of an autologous tumor preparation	53	53.4 years (range: 31-76)	26.0	NR	*IES* 19.8 (14.5)<br>*BSI* GSI 0.39 (0.32)	Patients with metastatic renal cell carcinoma; metastatic melanoma (Stage III or IV)
**Greer, 2019, USA**	Randomized clinical trial	Anxiety; depression; Quality of Life	Eligible patients were invited to participate in the study	Gastrointestinal cancer (24.7%), gynaecological cancer (19.2%), lung cancer (20.5%), breast cancer (11.0%), melanoma (6.8%), sarcoma (9.6%), genitourinary (6.8%), thyroid (1.4%); CBT group: intravenous chemotherapy (82.2%), oral chemotherapy	145 (72 CBT group, 73 control group)	56.5 (SD=11.3)	73.8	Median TSD: CBT group: 7.7 (range: 1.9- 23.3), control group: 7.5 (range: 2.0 - 24.3)	*HAM-A* 19.4 (9.0) versus 19.1 (7.6)<br>*HADS-A* 11.2 (2.4) versus 11.6 (3.0)<br>*HADS-D* 6.8 (3.1) versus 7.0 (3.4)<br>*PHQ-9* 9.2 (5.1) versus 9.3 (5.1) *FACT-G* 66.4 (12.3) versus 64.8 (16.3) for control versus CBT group	Patients with incurable cancer

				(5.5%), immunotherapy (13.7%), radiation (6.8%), surgery (6.8%); control group: intravenous chemotherapy (80.6%), oral chemotherapy (4.2%), immunotherapy (11.1%), radiation (6.9%), surgery (9.7%)						
**Greer, 2012, USA**	Pilot randomized controlled trial	Anxiety; Quality of Life	Recruitment via oncology clinicians, palliative care specialists, psychiatrists, or patients themselves through advertisements in the Massachusetts General Hospital (MGH) Cancer Center in Boston	Metastatic lung cancer (30.0%), metastatic pancreatic cancer (17.5%), metastatic colorectal cancers (15.0%), others (37.5%); CBT group (n=20): chemotherapy (80%), radiation (5%), ambulatory palliative care (35%), control group (n=20): chemotherapy (100%), radiation (10%), palliative care (30%)	40 (40 CBT group, 40 control group)	55.9 (SD=10.9, range: 31-81	70.0	Median TSD: CBT group: 6.6 months (range: 2.2-62.6), control group: 5.4 (range: 0.7-56.0)	*HAM-A* 24.5 (7.3) versus 24.4 (6.9) *MADRS* 23.4 (9.0) versus 22.4 (19.74) *HADS-A* 7.9 (4.5) versus 8.8 (4.6) *HADS-D* 7.7 (3.8) versus 9.1 (5.8) *IES* 32.3 (13.3) versus 36.1 (17.0) *FACT-G* 59.7 (14.4) versus 59.1 (16.9) EW 13.00 (5.1) versus 11.6 (5.5) for CBT group versus control group	Patients with terminal cancer
**Guo, 2017, China**	Longitudinal survey study	Depression	Recruitment via the Cancer Hospital of China Medical University, Liaoning Cancer Hospital & Institutes,	Metastatic breast cancer; chemotherapy	176	Median age: 49.5 (28-80)	100	NR	*Self-Rating Depression Scale (SDS)* major/medium depression risk 25.6%; minor depression symptoms 33.5%; not depressed 41.0%; no baseline SDS mean score for the total group reported	Patients with metastatic breast cancer
**Gupta, 2014, USA, France, Germany, Italy, Spain**	Cross-sectional survey study	Health-related Quality of Life; treatment satisfaction; family life	Convenience recruitment via panel recruiting, grassroots campaigns and newspaper advertising; patient support groups, physician referrals	Metastatic breast cancer; chemotherapy (n=191) or hormone therapy (n=169)	360	58.3 (SD=6.32)	100	Mean TSD: 4.5 years (SD: 5.0)	*FACT-G total* 56.4 (15.2) EW 12.0 (5.0) *FACT-B* 74.1 (19.2)	Metastatic breast cancer patients

**Khalaf, 2019, Canada**	Randomized controlled phase II trial	Health-related Quality of Life; depression symptoms	Recruitment not further specified	Metastatic castration-resistant prostate cancer; first-line treatment abiraterone and prednisone (n=101) or enzalutamide (n=101)	202	Median age: abitrante rone plus predniso ne arm: 72.9 (67.4-79.1); enzaluta mide arm: 77.6 (69.1–83.4)	0.0	NR	*FACT-P* 116 (105.7-130.1) versus 114 (100.0-128.0)<br>*PHQ-9* 2 (1-5) versus 4 (1-7) for the arbiterone group versus enzalutamide group	Patients with metastatic castration-resistant prostate cancer
**Kissane, 2004, Australia**	Cross-sectional survey study	Quality of Life; depression; anxiety; coping	Recruitment from oncology services in seven general metropolitan hospitals and a range of private practitioners	Metastatic breast cancer; chemotherapy (plus further hormonal therapy, chemotherapy or radiotherapy)	200	51.0 (SD=9)	100	52 months (SD: 41)	*EORTC-QLQ-C30*<br>*HADS*<br>NR<br>*MAC*<br>NR<br>*MCMQ*<br>NR	Patients with metastatic breast cancer
**Lacey, 2019, Australia**	Pre-post-test feasibility cohort study (pilot study)	Quality of Life; depression; anxiety	Recruitment via referral by treating oncologists or nurses from a single institution. Control group includes individuals who were eligible for the study but declined the supportive care intervention (	Metastatic melanoma; immunotherapy (pembrolizumab) group (n=13) versus control group (n=15)	28	Median age: 66.0 (42-85)	43.0	Median TSD: 8 years (0.8-30.0); median TSD of metastatic disease: 2.8 years (range: 0.18–11.0)	*FACT-G* Total 89.5 (9.7) EW 20.2 (2.6) *ESAS* (intervention versus usual care group) depression 0.5 versus 0.4 anxiety 0.9 versus 0.5 *HADS-A* 3.0 (2.3) *HADS-D* 4.3 (3.4) *HADS-Distress* 7.3 (4.9)	Patients with metastatic melanoma
**Lai-Kwon, 2019, Australia**	Cross-sectional survey study	Quality of Life; anxiety; fear of recurrence or progression or death	Recruitment via weekly review of the Melanoma Service clinic lists	Metastatic melanoma; immune checkpoint inhibitors (ICI) (n=69) or BRAF and MEK inhibitors (BMi) (n=36); ICI (n= 39): 57% were receiving ongoing treatment, 17 ceased due to toxicity, BMi (n=31): 85% were receiving ongoing treatment, and 4 ceased due to toxicity	105	Median age: 62.0 (24-88)	43.0	Mean TSD: ≤12 months (20%), 12-24 months (29%), >24 months (51%)	No outcomes of validated measures were reported	Long term responders; metastatic melanoma patients

**McFarland, 2019, USA**	Cross-sectional survey study	Anxiety; depression; distress	Recruitment via treating staff members, not further specified	Adenocarcinoma non-small cell lung cancer (71.8%), small cell lung cancer (16.5%), squamous cell carcinoma (6.4%), unspecified type of lung cancer (4.6%); chemotherapy (45.2%), immunotherapy (33.7%), targeted therapies (21.2%), and 5.5% missing treatment	109	65.9	62.4	15.4 months (SD: 17.3)	*HADS-A* 5.39 (3.94) *HADS-D* 4.9 (3.7) *DT&PL* 3.91 (2.96)	Patients with metastatic lung cancer undergoing treatment; patients with stage IV lung cancer who were receiving anticancer treatment
**McFarland *(Shaffer)*, 2019, USA**	Cross-sectional survey study	Anxiety; Depression	Recruitment via treating staff members, not further specified	Adenocarcinoma non-small cell lung cancer (71.8%), small cell lung cancer (16.5%), squamous cell carcinoma (6.4%), unspecified type of lung cancer (4.6%); chemotherapy (45.2%), immunotherapy (33.7%), targeted therapies (21.2%), and 5.5% missing treatment	109	65.9	62.4	15.4 months (SD: 17.3)	*HADS-A* 5.39 (3.94) *HADS-D* 4.9 (3.7)	Metastatic lung cancer patients receiving immunotherapy, targeted therapies, or anti-cancer treatments
**Mir, 2018, France**	Case-control study	Depression; mood changes	Consecutive sampling;	Metastatic breast cancer; everolimus and hormone therapy (n=20), or hormone therapy (n=20)	40	Everolimus plus hormone therapy: 62.2 (51.1-74.6), hormone therapy: 62.9 (51.6-72.0)	100	NR	*BDI* 7.15 (5.48) versus 7.15 (4.36) *MADRS* 14.0 (4.09) versus 14.6 (3.6) for hormone therapy group versus everolimus plus hormone therapy group	Post-menopausal patients with metastatic, estrogen receptor positive breast cancer resistant or refractory to aromatase inhibitors; patients with metastatic breast cancer treatment
**Miyake, 2014, Japan**	Case-control study	Health-related Quality of Life	Recruitment not further specified	Metastatic renal cell carcinoma; tyrosine kinase inhibitors	240	Median age: 62.5 (37-86)	22.0	NR	*SF-36* overall score not reported	Metastatic renal cell carcinoma patients treated with TKIs; patients with

				(sorafenib, sunitinib, axitinib)						metastatic cancer; patients with metastatic diseases who are incurable and have a limited life expectancy
**Moscetti, 2017, Italy**	Multi-center prospective observational study	Health-related Quality of Life	Recruitment via four Italian cancer centers	Metastatic breast cancer; eribulin mesylate	50	64.0 (31-85)	100	NR	*ESAS* 32.6 (19.4) *FACT-G* 56.7 (12.9) *FACT-B* 78.1 (16.5)	Patients with advanced breast cancer
**Mu, 2004, China**	Prospective pre-post cohort study	Quality of Life	Recruitment via Peking union medical college hospital	Squamous-cell carcinoma (32.3%), adenocarcinoma (64.5%); gefitinib (EGFR-TKI); at least one platinum-based regimen and most had received more than two regimens (different combinations including platinum, taxane, docetaxel and gemcitabine)	31	Median age: 64.0 (28-85)	42.0	NR	*EORTC QLQ-C30 QL* 36.0 (24.7) EF 67.67 (28.39)	Patients with advanced or metastatic non-small-cell lung cancer
**Mulder, 2014, The Netherlands**	Cross-sectional survey study	Mood; psychological wellbeing	Recruitment of patients via treating specialists; recruitment of controls via acquaintances of the patients and by advertisements in local papers	Metastatic renal cell carcinoma (mRCC) or gastrointestinal stromal tumors (GIST); VEGFR sunitib or sorafenib	30	60.0 (38-81)	10.0	NR	*SCL-90-R* Total 123.10 (24.13) Anxiety 13.07 (3.10) Depression 23.87 (6.31) *BDI-II* 9.87 (5.76)	Patients with metastatic renal cell carcinoma
**O'Reilly, 2020, UK**	Cross-sectional survey study	Health-related Quality of Life	Recruitment via the melanoma clinic in the outpatients department at the Royal Marsden NHS foundation trust of all eligible patients	Metastatic melanoma; immune checkpoint inhibitors: ipilimumab (19%), pembrolizumab (37%), nivolumab (21%), ipilimumab plus nivolumab (14%), blinded clinical trial (8%)	73	Median age: 65.0 (22-86)	36.0	NR	*SF-36* GH 65.3 RE 78.5	Metastatic melanoma survivors; patients with a diagnosis of metastatic melanoma who achieved a durable response to an ICI
**Osoba, 2002, USA**	Randomized controlled trial	(Health-related) Quality of Life	Secondary analysis of prospective longitudinal questionnaires as part of an RCT	Metastatic breast cancer; chemotherapy with (n=208) or without trastuzumab (n=192)	400	53.0 (25-76) versus 52 (25-72) for trastuzu	100	NR	*EORTC-QLQ-QL* 59 (26) versus 58 (25) EW 67 (23) versus 66 (26) for trastuzumab+chemoth	Patients with metastatic breast cancer

						mab+chemotherapy versus chemotherapy alone			erapy versus chemotherapy alone	
**Oswald, 2019, USA**	Cross-sectional survey study	Optimism	Recruitment via the urology and medical oncology clinics at VUMC and from the ZERO website	Metastatic prostate cancer; systemic treatment not further specified	100	68.3 (SD=8.7)	0.0	NR	*Lot-R* 19.6 (4.31)	Patients with (incurable) metastatic prostate cancer
**Goebell, 2014, Germany**	Cross-sectional survey study	Health-related Quality of Life	Recruitment via a multicentre, prospective, noninterventional clinical mRCC Registry. (i.e. the mRCC registry, NCT00610012)	Advanced or metastatic renal cell carcinoma (RCC); sunitinib (n=50), sorafenib (n=15), temsirolimus (n=16), bevacizumab in combination with interferon alpha (n=11), everolimus or interferon alpha (n=6)	98	Median age: 70.5	27.4	NR	*FACT-G* 73.5 (18.3)	Patients with advanced or metastatic renal cell carcinoma
**Parente, 2017, Australia**	Results from a randomized controlled trial	Quality of Life; happiness; mental health; coping; relationships; self-worth	Recruitment from eighteen Australian sites enrolled in a worldwide early access program study	Metastatic prostate cancer; cabazitaxel after docetaxel-based treatment	104	Median age: 70.0 (43-89)	0.0	Median TSD: 53.93 months (36.5-82.4); median time since mCRPC diagnosis: 19.97 months (11.2-34.2)	*Australian Quality of Life (AQoL-8D) questionnaire* 0.7 (0.2) psychological subscale 0.4 (0.2)	Patients with metastatic castration-resistant prostate cancer (mCRPC)
**Patasius, 2019, Lithuania**	Retrospective cohort study	Suicide	Prostate cancer cases diagnosed stage III or IV were extracted from the Lithuanian population-based Cancer Registry database; ICD-10-codes were identified	Prostate cancer stage III (86.3%) or stage IV (13.7%); androgen deprivation therapy	5156	NR	0.0	NR	32 suicides were reported. No validated questionnaires were reported	Patients with advanced prostate cancer
**Paterson, 2018, Scotland**	Pilot randomized controlled trial	Depression; anxiety; Health-related Quality of Life; self-efficacy	Recruitment via four hospitals in Scotland	Metastatic prostate cancer; androgen deprivation therapy	38	74.9 (SD=8.2, range: 60-86) in the	0.0	7-56 months	*Self-management Self-Efficacy Scale (SES)* 3.5 (0.8); *EORTC-QLQ-C30* QL 73.5 (22.2) EF	Patients with metastatic prostate cancer on hormonal treatment

						intervention group; 77.5 years (SD: 6.2; 66-93) in the standard care group			87.5 (15.6) *HADS-A 2.9 (3.4) HADS-D* 3.6 (3.6)	
**Peters, 2014, The Netherlands**	Cross-sectional survey study	Coping; fear of disease progression; social support and interactions; depressive mood related to fatigue	Recruitment via the department of Medical Oncology of a university and a regional hospital in the south eastern part of the Netherlands via preselection by the treating physician	Advanced, incurable cancer: 30% breast cancer, 30 gastrointestinal cancer, 11% urogenital cancer, 9.5% gynecological cancer, 9.5% bone and soft tissue cancer, 10% other cancers; palliative (symptom reducing or life prolonging) therapy: 53% chemotherapy, 15% oral targeted therapy, 15% hormonal therapy, 14% chemotargeted therapy, 1% radiotherapy, 1% chemo-radiotherapy, 1% no treatment	137	59.0 (30-79)	61.0	NR	*Subscale acceptance of the Illness Cognition Questionnaire* 17 (4.3) *HADS-A* 6 (4.3) *HADS-D* 4 (3.6)	Patients with advanced incurable disease; patients on active treatment for various incurable cancers
**Peters, 2016, The Netherlands**	Longitudinal prospective cohort study	Non-acceptance of having incurable cancer; anxiety; depressive mood; social support	Recruitment via the department of Medical Oncology of a university and a regional hospital in the south eastern part of the Netherlands via preselection by the treating physician	Advanced, incurable cancer: 30% breast cancer, 30 gastrointestinal cancer, 11% urogenital cancer, 9.5% gynecological cancer, 9.5% bone and soft tissue cancer, 10% other cancers; palliative (symptom reducing or life prolonging) therapy: 53%	137	59.0 (30-79)	61.0	NR	*Subscale acceptance of the Illness Cognition Questionnaire* median score 17 *HADS-A* median score 5 *HADS-D* median score 4	Patients with advanced cancer receiving palliative cancer treatment; patients with incurable cancer (while on treatment)

				chemotherapy, 15% oral targeted therapy, 15% hormonal therapy, 14% chemotargeted therapy, 1% radiotherapy, 1% chemo-radiotherapy, 1% no treatment						
**Rouanne, 2013, France**	Prospective cohort study	Health-related Quality of Life; depression	Consecutive sampling; recruitment via phase I oncology trials at Institut Gustave Roussy (Villejuif, France)	Prostate cancer (n=3), testis cancer (n=1), renal cell carcicoma (n=1), endometrial and cervical (n=6), breast cancer (n=10), ovarian cancer (n=4), pancreas cancer (n=2), pleural mesothelioma (n=2), gastric cancer (n=3), colorectal cancer (n=9), non-small cell lung cancer (n=4), small cell lung cancer (n=5), head and neck cancer (n=6), sarcoma (n=1), melanoma (n=6); targeted anticancer drugs in phase I clinical trials	63	54.7	39.7	Mean TSD: 41 months	*SF12; BDI*	Advanced or metastatic cancer patients referred to Phase I oncology trials; patients with advanced or metastatic disease included in Phase I oncology trials; advanced cancer patients
**Schuurhuizen *(Braamse, Konings)*, 2019, The Netherlands**	Secondary analysis data from a randomized clinical trial	Health-related Quality of Life; depression; anxiety; psychological distress; Quality of Life	Analyses are based on 349 outpatients, recruited for a RCT throughout 16 hospitals in the Netherlands	Metastatic colorectal cancer; starting with first-line systemic palliative chemotherapy	349	66.1 (SD=10.2)	35.8	Age at diagnosis: 23-83	*HADS; DT&PL; EORTC-QLQ-C30*	Patients with metastatic colorectal cancer (receiving first line systemic treatment)
**Schuurhuizen *(Braamse, Beekman)*, 2019, The Netherlands**	Multicentre, clustered randomized trial	Quality of Life; depression; anxiety	Recruitment via the medical oncology departments of 16 participating hospitals in the Netherlands	Metastatic colorectal cancer; starting with first-line systemic palliative chemotherapy	349	66.1 (SD=10.2)	35.8	TSD: < 1.5 months: 33.2%, 1.5-10 months: 32.9%, >10 months: 33.2	*HADS* 9.52 (6.6) *EORTC-QLQ-C30* QL 63.0 (21.8) EF 77.1 (18.9)	Patients with metastatic colorectal cancer
**Selvi, 2020, Germany**	Prospective cohort study	Health-related Quality of Life; post-traumatic	Recruitment not further specified	Metastatic prostate cancer; luteinizing hormone-releasing	60	69.83 (SD=5.3)	0.0	NR	*Post-traumatic stress disorder-civilian version*	Patients with metastatic prostate cancer

		stress disorder (PTSD)		hormone (LHRH) analogue treatment (n=60)					*(PTSD-CV); SF-36* Baseline results NR	
**Sherliker, 2000, UK**	Feasibility study/mixed-methods	Coping; psychological well-being; mental adjustments; mood symptoms	Recruitment via phase I clinical trials at the ICRF Medical Oncology Unit in Oxford, UK	Advanced metastatic cancer including lung cancer, breast cancer, colon cancer and kidney cancer; combination of fluorouracil, folinic acid and interferon (n=8) or experimental drugs (n=2)	10	45-71	60.0	NR	*MAC* Anxious preoccupation 23.3 (4.0) Fatalism 19.3 (3.3) Fighting spirit 50.4 (6.2) Helpless/hopeless 9.9 (3.1) *HADS-A* 5.1 (1.5) *HADS-D* 2.9 (2.2) *POMS* Baseline results NR	Patients with advanced cancer; patients with metastatic cancer; patients with advanced metastatic cancer
**Shin, 2016, USA**	Cross-sectional survey study	Quality of Life; depression; anxiety	Consecutive recruitment via a trained research assistant at the ambulatory care clinic at the Massachusetts General Hospital Cancer Center through electronic medical records	Metastatic breast cancer; receiving endocrine therapy (n = 40) or chemotherapy (n=100)	140	66.0 (SD=11.3) for endocrine therapy, 58.6 (SD: 11.8) for chemotherapy	100	NR	*FACT-B* 111.6 (18.4) versus 104.9 (20.0) EW 17.9 (3.6) versus 17.0 (4.6) *HADS-A* 4.8 (3.4) versus 5.7 (4.1) *HADS-D* 3.0 (3.1) versus 4.5 (3.7) for endocrine therapy versus chemotherapy respectively	Patients with metastatic breast cancer
**Steffen McLouth, 2020, USA**	Cross-sectional survey study	Health-related Quality of Life	Purposive sampling; recruitment via an academic medical center	Metastatic non-small cell lung cancer (NSCLC); immunotherapy or chemoimmunotherapy	60	62.5 (SD=9.3)	60.0	NR	*EORTC-QLQ-C30 QL* 62.6 (21.5) EF 79.7 (20.1)	(Metastatic) non-small cell lung cancer patients receiving immunotherapy; metastatic lung cancer patients treated with immunotherapy
**Udupa, 2017, India**	Longitudinal survey study	Quality of Life	Recruitment via the Cancer Institute, Adyar, Chennai	Metastatic adenocarcinoma; tyrosine kinase inhibitors (n=20), or various platinum-based doublet chemotherapy (n=23)	43	58.4 versus 59.1 for TKI versus chemotherapy respectively	30.2	NR	*Cancer Institute-QOL Questionnaire (CI-QOL-Q)* TKI: 128 (NR)	Metastatic lung cancer patients; advanced lung cancer
**Van Gool, 2008, The Netherlands**	Longitudinal survey study of a subset of patients of a larger study	Anxiety; depression	Consecutive sampling; recruitment of the second half of included patients in a larger study	Metastatic renal cell carcinoma; conventional short-acting IFN-alpha	24	Median age: 60.5 (47–72)	33.3	NR	*SCL-90* 110.7 (15.5) Anxiety 12.3 (2.0) Depression 21.4 (4.4)	Patients with metastatic renal cell cancer
**Wang, 2018, China**	Prospective cohort study	Quality of Life; depression; anxiety	Consecutive sampling via the Department of Urinary Surgery in	Metastatic renal cell carcinoma;	127	54.9 (SD=11.2)	20.5	NR	*HADS-A* 5.8 (3.2) *HADS-D* 6.4 (3.8)	Patients with metastatic renal cell carcinoma

			Harbin Medical University Cancer Hospital	interferon-alpha (IFN-α) treatment					*EORTC-QLQ-C30 QL* 64.0 (14.3)	
**Wood, 2017, USA, Europe**	Cross-sectional survey study	Quality of Life; treatment satisfaction	Convenience sampling; Recruitment via participating physicians who recruited 8-10 patients from the United States, France, Germany, Italy, Spain, and the United Kingdom	HR+/HER2- advanced/metastatic breast cancer: stage IV: 83%, stage IIIB/C: 17%; chemotherapy only: 41.3%; endocrine therapy only: 39.6%; chemotherapy and/or endocrine combination therapies with or without targeted agents: 19.1%	739	65.2 (SD=10.6)	100	NR	*FACT-B* 85.9 (19.7) EW 13.1 (4.5) *FACT-G* 61.8 (15.8)	Patients with advanced or metastatic breast cancer
**Wyatt, 2012**, USA**	Secondary analysis of data from a randomized clinical	Health-related Quality of Life	Recruitment via 13 community-based medical oncology sites in the Midwest of the USA	Advanced breast cancer with distant metastases; chemotherapy and/or hormonal therapy	298	56.8 (11.2)	100	NR	*SF-36* 53.6 (27.5*) State Anxiety Scale* 35.8 (11.9) *CES-D* 14.7 (10.3)	Patients with advanced breast cancer
**Yang, 2018, Taiwan**	Longitudinal survey study	Quality of Life	Quasi-random sampling; recruitment via the outpatient departments of NCKUH	Recurrent or newly diagnosed EGFR-mutation-positive advanced non-small cell lung cancer (NSCLC); Gefitinib, erlotinib, afatinib	344	63.7 (SD=11.2), 61.9 (SD=12.8), 60.8 (SD=10.2) for gefitinib, erlotinib, afatinib respectiv ely	63.2, 55.6, 59.6 for gefitinib, erlotinib , afatinib respecti vely	NR	*EQ-5D; WHOQOL-BREF baseline means NR*	Patients with advanced non-small cell lung cancer
**Adamowicz, 2020, Poland**	Prospective cohort study	Quality of Life	Recruitment via two centres in Poland	Advanced metastatic breast cancer; palliative chemotherapy (40%), hormone therapy (34%), antiHER2-therapy (26%)	351	62 (SD=9.8)	100	NR	*EORTC-QLQ-C30 QL* 60.9 (23.7) EF 68.4 (25.7)	Palliative patients; patients with advanced, metastatic breast cancer
**Bjelic-Radisic, 2020, Austria**	Randomized controlled trial (ABCSG-29 Posytive Trial)	Quality of Life	Recruitment via fifteen centres in Austria	Metastatic breast cancer; chemotherapy or endocrine	79	Median age: 62.8	100	NR	*EORTC-QLQ-C30 QL* 47.8 (4.3) versus 61.6 (4.2) EF 58.1 (4.3) versus 62.3 (4.1) for primary surgery versus primary	Patients with primary metastatic breast cancer

									systemic therapy respectively	
**Boevé, 2021, The Netherlands**	Randomized controlled trial (HORRAD)	Health-related Quality of life	Recruitment via twenty-eight centres in the Netherlands	Primary bone metastatic prostate cancer; androgen deprivation therapy (ADT) and radiotherapy (RT)versus ADT alone	432	67.0 (62-71) versus 66.5 (61-71) for ADT + RT versus ADT alone respectively	0.0	NR	*EORTC-QLQ-C30 QL* 70.2 (22.8) versus 70.0 (22.6) EF 74.4 (21.2) 73.6 (22.5) for ADT+RT versus ADT alone respectively	Patients with primary bone metastatic prostate cancer
**Cicin, 2020, Turkey**	Multicenter, non-interventional longitudinal study	Quality of Life	Recruitment via twenty-eight centers in twelve regions of Turkey	Metastatic renal cell carcinom; axitinib (75.5%) or everolimus (24.5%)	102	Median age: 61.0 (SD=12, 24-83)	27.5	Median TSD: 27.5 months (4-201)	*EQ-5D-3L* 0.49 (0.14-1) versus 0.48 (-0.74-1) for axitinib versus everolimus respectively	Advanced renal cell carcinoma patients; metastatic renal cell carcinoma
**Claessens, 2020, The Netherlands**	Cross-sectional analysis of data from an ongoing real-world multi-center cohort study (SONABRE registry)	Quality of Life	Recruitment from four hospitals participating in the SONABRE registry, by invitation by treating oncologists when visiting the outpatient ward	Advanced breast cancer (stage IV); receiving endocrine therapy (with or without targeted therapy) (51%), chemotherapy (with or without targeted therapy (37%), targeted therapy alone (5%), none (7%)	92	65% <65, 35% ≥65	100	NR	*EQ-5D-3L baseline mean NR*	Advanced breast cancer patients
**Davie, 2020, France, Germany, Italy, Spain, UK**	Multinational, real-world, point-in-time, patient record-based survey study	Health-related Quality of Life	Random recruitment via oncologists identified from publicly available lists of healthcare professionals	Advanced breast cancer (stage IIIb (1%), stage IIIc (19%), stage IV (79%)); endocrine therapy with or without other systemic treatment(s)	252	67.1 (SD=10.8)	100	NR	*EORTC-QLQ-C30 QL* 50.9 (24.7) EF (61.9 (26.2) *EQ-5D-3L* 0.69 (0.28)	Advanced disease; patients with advanced breast cancer
**Davies, 2020, UK**	Phase III, open-label, parallel-group study (GALLIUM-study)	Health-related Quality of Life	Recruitment not further specified	Advanced follicular lymphoma (grade 1-3a); obinutuzumab versus rituximab plus chemotherapy	1202	Median age: 60 (26-88) versus 58 (23-85) for obinutuz umab	52.9% versus 53.4% for obinutu zumab versus rituxima	Median TSD: 1.5 months (0.1-121.6) versus 1.4 months (0.0-168.1) for	*FACT-G* EW 17.6 versus 17.9 for obinutuzumab versus rituximab plus chemotherapy respectively	Patients with advanced follicular lymphoma

						versus rituximab respectively	b respectively	obinutuzumab versus rituximab respectively		
**Fasching, 2020, Germany**	Phase III, randomized, double-blind, placebo-controlled trial (MONALEESA-3)	Health-related Quality of Life (time to definitive 10% deterioration from baseline in HRQoL)	Recruitment via 174 study sites in 30 countries	HR+/HER2- advanced breast cancer (98.8% stage IV); ribociclib and fulvestrant or placebo and fulvestrant	726	Median age: 63.0 (31-89) versus 63.0 (34-86)	100	NR	*EORTC-QLQ-C30* 65.5 (19.1) versus 68.4 (18.5)	Patients with advanced breast cancer
**Fizazi, 2020, 13 European countries**	Secondary analysis of data from a phase IV, randomized, multicentre, open-label study (CARD study)	Quality of Life	Recruitment from 62 clinical sites across 13 European countries	Metastatic prostate cancer; cabazitaxel (n=129) versus abiraterone or enzalutamide (n= 126)	255	Median age: 70 (65-76) versus 71 (64-75) for cabazitaxel versus arbiterone or enzalutamide	0.0	NR	*FACT-G* 73.7 (16.5) versus 75.1 (16.6) EW 16.2 (4.5) versus 16,8 (4.7) *EQ-5D-5L VAS* 65.8 (20.4) versus 66.3 (18.5) for cabazitaxel versus abiraterone or enzalutamide	Patients with metastatic prostate cancer
**Harbeck, 2020, 30 countries**	Phase III, randomized, double-blind, placebo-controlled trial (MONALEESA-7)	Health-related Quality of Life	Recruitment via 188 centres in 30 countries	Advanced breast cancer; ribociclib (49.9%) or placebo (50.1%)	672	43.0 (25-58) versus 45.0 (29-58)	100	NR	*EORTC-QLQ-C30 EQ-5D-5L baseline means NR*	Patients with advanced breast cancer
**Hollen, 2020, USA**	Randomized controlled trial	Treatment-related regret	Recruitment via five hospitals in the USA	Advanced non-small lung cancer; chemotherapy or a checkpoint inhibitor	160	Age ≥70: 33% versus 27% for patients with regret versus without regret	33% versus 46%	NR	*Decision regret scale (DRS) Decisional conflict scale (DCS)* 15.6 (12.9-18.2); versus 17.9 (15.7-20.3)	Patients with advanced lung cancer
**Joly, 2020, France**	Multicenter prospective observational cohort study (FUJI)	Quality of Life	Prospective sampling; recruitment via oncologists from various centers in France	Metastatic castration-resistant prostate cancer; cabazitaxel	60	Median age: 72.0 (69-78)	0.0	NR	*FACT-P* 93.3 (18.3) EW 16.0 (4.8) *FACT-G* 66.7 (13.5)	Patients with metastatic castration-resistant prostate cancer

**Joseph, 2020, USA**	Prospective observational study	Quality of Life	Consecutive sampling; recruitment via nine academic and satellite centers in the US	Advanced melanoma; pembrolizumab (PEMBRO) (n=225) or ipilimumab (IPI) plus nivolumab (NIVO) (n=187)	412	63.8 (8.9) versus 61.5 (8.3) for PEMBRO versus IPI plus NIVO	46.2 versus 39.6 for PEMBRO versus IPI plus NIVO	NR	*EORTC-QLQ-C30 QL* 58.8 (22.3) versus 59.5 (22.6) *EQ-5D-5L VAS* 65.0 (21.4) versus 67.7 (21.2) for PEMBRO versus IPI+NIVO	Patients with advanced melanoma
**Lee, 2020, South Korea**	Multicenter, prospective, open-label, randomized phase ll trial	Quality of Life	Recruitment via fourteen academic institutions in South Korea	Metastatic breast cancer; palbociclib plus endocrine treatment (n=92) versus capecitabine (n=86)	178	Median age: 44.0 (28-58)	100	NR	*EORTC-QLQ-C30 QL* 65.2 (20.8) versus 57.9 (22.3) EF 74.8 (18.7) versus 70.2 (21.6) for palbociclib plus endocrine treatment versus capecitabine	Metastatic breast cancer patients
**Lu, 2021, Korea, Taiwan, Thailand**	International, multicenter, open-label, parallelgroup, phase Ib, dose, de-escalation study	Quality of Life; anxiety; depression	Recruitment via fifteen centers in Hong Kong, Republic of Korea, Taiwan, and Thailand	Locally advanced or metastatic breast cancer (96.6%); tamoxifen and goserelin with either alpelisib or buparlisib	39	45.3	100	NR	*EQ-5D-5L PHQ-9 General Anxiety Disorder-7 Scale (GAD-7)* for alpelisib versus buparlisib baseline means NR	Patients with advanced breast cancer
**Lynch, 2020, Australia**	Feasibility study	Fear of cancer recurrence	Recruitment via the research staff at melanoma outpatient appointments at two hospitals in Australia	Metastatic melanoma; immunotherapies and/or targeted therapies	61	61.4 (SD=11.6)	33.0	NR	*FCRI-SF* 14.7 (7.77) *FoP-Q-SF* 25.4 (10.12)	Survivors with advanced cancer/malignancies
**Marschner, 2020, Germany**	Cohort study; data analysis of four prospective non-intervention, multicenter registries	Health-related Quality of Life, associated with disease progression	Recruitment via 203 sites in Germany	Metastatic breast cancer (20.1%), metastatic pancreatic cancer (34.9%), metastatic lung cancer (14.7%), metastatic colorectal cancer (30.3%); systemic palliative first-line treatment	2314	Median age: metastatic breast cancer: 61.6 (26.4-90.1), metastatic pancreatic cancer: 70.0 (39.0-93.0), metastatic lung cancer: 65.9 (28.4-88.2),	Metastatic breast cancer: 100, metastatic pancreatic cancer: 43.6, metastatic lung cancer: 34.6, metastatic colorectal cancer: 35.3	NR	*FACT-G; EORTC-QLQ-C30; EORTC-QLQ-C15-PAL; HADS baseline means NR*	Patients with metastatic cancer

						metastatic colorectal cancer: 66.9 (26.9-92.1)				
**Marshall (Given), 2019, USA**	Secondary analysis of data from a randomized controlled trial	Treatment beliefs	Recruitment via six National Cancer Institute-designated comprehensive cancer centers	Advanced cancer; oral antineoplastic agents	60	63.8 (SD=10.3)	62.0	NR	*Beliefs about Medicine Questionnaire-Specific (BMQ)*	Patients with (advanced) stage cancer
**McFarland, 2020, USA**	Cross-sectional survey study	Anxiety; depression	Recruitment not further specified	Stage IV lung cancer; chemotherapy (46.1%), immunotherapy (31.5%), targeted therapy (20.8%), missing (11.4%)	96	66.1 (SD=9.2)	61.1	NR	*HADS-A* 5.4 (3.9) *HADS-D* 4.8 (3.5)	Patients with metastatic lung cancers
**Murray, 2020, USA**	Pilot study	Quality of Life	Recruitment not further specified	Advanced or metastatic prostate cancer; androgen deprivation therapy	27	40-85	100	NR	*SF-12 (v2) QL* 44.2 RE 47.8 MH 53.0; *FACIT-P* EW 18.4	Men with advanced and metastatic cancer
**Ryoo, 2020, USA**	Exploratory analysis of data from a randomized placebo-controlled and double-blind study (KEYNOTE-240)	Health-related Quality of Life	Secondary analysis of data via the KEYNOTE-240-study	Advanced hepatocellular carcinoma; pembrolizumab (n=271) versus placebo (127)	398	NR	NR	NR	*EORTC-QLQ-C30* 70.4 (20.1) versus 68.9 (21.5) for pembrolizumab versus placebo respectively	Patients with advanced disease
**Slama, 2020, Czech Republic**	Single-center unblinded randomized controlled trial (PALINT)	Quality of Life	Recruitment via primary oncologists from a single institute	Advanced cancers: head and neck tumours (5.0% versus 0.0%), esophagus and stomach tumours (30.0% versus 24.2%), lung tumours (20.0% versus 21.2%), pancreas tumours (23.3% versus 34.8%), colon tumours (21.7% versus 19.7%) for	126	61.1 (9.8) versus 63.5 (10.4) for intervention versus control group respectively	38.3 versus 42.4 for intervention and control group respectively	NR	*EORTC-QLQ-C30 QL* 58.6 (53.9-63.3) versus 54.2 (49.4-58.9) EF 82.8 (77.9-87.7) versus 84.1 (79.8-88.4) *HADS* 10 (0-29) versus 9 (0-32) *HADS-A* 4 (0-15) versus 4 (0-15) *HADS-D* 6 (0-18) versus 5 (0-17) for intervention and control group respectively	Patients with (newly diagnosed) advanced cancer; patients with advanced solid tumors

				intervention and control group respectively						
**Stenzl, 2020, Argentina, Australia, Belgium, Canada, Chile, Denmark, Finland, France, Germany, Israel, Italy, Japan, New Zealand, Poland, Romania, Russia, Slovakia, South Korea, Spain, Sweden, Taiwan, the Netherlands, UK, USA**	Randomized, double blind, placebo-controlled, phase III study (ARCHES; NCT02677896)	Health-related Quality of Life	Recruitment via twenty-four countries, not further specified	Metastatic hormone-sensitive prostate cancer; enzalutamide (49.9%) or placebo plus ADT (50.1%)	1150	70.0 (46-92) versus 70.0 (42-92) for enzalutamide versus placebo respectively	0.0	NR	*FACT-P* EW 18.4 (4.2) versus 18.2 (4.1) *FACT-G 80.5 (14.8) versus 80.23 (14.0) EQ-5D-5L VAS* 74.4 (17.1) versus 74.2 (16.9) for enzalutamide versus placebo	Patients with metastatic hormone-sensitive prostate cancer
**Taarnhoj, 2020, Denmark**	Longitudinal survey study with data from two prospective clinical studies	Quality of Life	Recruitment not further specified	Advanced bladder cancer (67% metastatic disease); chemotherapy or immunotherapy	79	68 (35.1)	19.0	NR	*EORTC-QLQ-C30 QLQ-BLM30 HADS*	Patients with advanced disease
**Thom, 2020, USA**	Cross-sectional survey and medical record review	Quality of Life	Recruitment via an institutional patient database	Advanced melanoma; Immune checkpoint inhibitors	106	63.0 (SD=12.5)	43.0	NR	*EORTC-QLQ-C30* QL 84.0 (19.5) EF 84.6 (16.7)	Cancer survivors
**Verrill, 2020, UK**	Cross-sectional survey study	Health-related Quality of Life	Mixed approach recruitment via physician referral from fourteen secondary and tertiary care centres across England	Metastatic breast cancer; treatment not further specified	102	55.3 (11.2)	100	30.0 months (37.0)	*EQ-5D-5L VAS* 65.8 (22.9) *FACT-G; FACT-B 92.2 (27.4)* EW 15.2 (6.0) *FACT-G* 71.1 (22.4)	Patients with metastatic disease
**Westdorp, 2020, The Netherlands**	Longitudinal survey study (part of a phase IIa trial)	Health-related Quality of Life	Recruitment via a RCT	Castration-resistant prostate cancer; dendritic cell vaccination therapy	21	67 (53-82)	0.0	NR	*EORTC-QLQ-C30 QL* 75.4 (16.6) EF 75.8 (20.2) *BDI 1.3 (1.8)*	Advanced prostate cancer patients

**Long, 2016, Australia**	Phase III, randomized, double-blind study (CheckMate 066)	Health-related Quality of Life	Recruitment via eighty centers in Europe, Israel, Australia, Canada, and South America	Treatment-naïve advanced melanoma; nivolumab (50.2%) or dacarbazine (49.8%)	418	Median age: 65.0 (18-87)	41.1	NR	*EORTC-QLQ-C30* QL 68.9 (20.2) versus 66.2 (25.1) *EQ-5D VAS* 70.9 (19.9) versus 69.1 (21.8) for nivolumab versus dacarbazine respectively	Patients with treatment-naïve advanced melanoma
**El-Jawahri, 2014, USA**	Cross-sectional survey study	Quality of Life; mood symptoms	Consecutive sampling; recruitment via the patient database of the Massachusetts General Hospital Gastrointestinal Cancer Center by a trained research assistant with obtained permission from the treating oncologist	Incurable non-colorectal gastrointestinal cancer (78% hepatobiliary/pancreatic cancer, 22% gastroesophageal cancer); chemotherapy	50	66.9 (SD=10.4)	28.0	NR	*HADS-A* 3.5 (3.5) versus 5.4 (3.4) *HADS-D* 4.5 (3.7) versus 5.4 (2.9) *FACT-G* 80.9 (13.5) versus 73.1 (11.8) EF 18.2 (3.4) versus 15.0 (4.4) for curable prognostic perception versus not curable prognostic perception respectively	Patients with advanced cancer; patients with advanced, incurable cancers
**Poort, 2019, USA**	Secondary analysis of baseline data from a randomized controlled trial	Anxiety; depression; Quality of Life	Recruitment via the outpatient oncology clinics at Massachusetts General Hospital (MGH) Cancer Center in Boston, Massachusetts and two satellite clinics by study staff with permission from clinical oncologists to approach eligible patients	Hematologic malignancies (33%), non-small cell lung cancer (18%), breast cancer (14%), glioma (14%), sarcoma (8%), gastrointestinal (4%), melanoma (4%), genitourinary (4%), targeted therapy (66%) or oral chemotherapy (33%)	180	53.3 (SD=12.9)	53.3	NR	*HADS-A* 4.3 (3.3) versus 8.0 (4.1) *HADS-D* 3.1 (2.5) versus 7.3 (3.0) *FACT-G* 86.7 (12.8) versus 67.1 (11.7) EW 18.5 (4.0) versus 15.5 (4.8) for no-mild fatigue versus moderate-severe fatigue respectively	Patients with advanced cancer
**Greer, 2008, USA**	Longitudinal survey study with data from a single-arm, prospective feasibility study	Quality of life; anxiety; depression	Recruitment via a single-arm, prospective study from the Massachusetts General Hospital Palliative Care Team	Advanced non-small cell lung cancer (stage IV 94%, stage IIIb 6%); chemotherapy	50	65.7 (SD=8.7)	60.0	NR	*FACT-L* 94.4 (16.4) *HADS-A HADS-D baseline means NR*	Patients with advanced non-small-cell lung cancer; patients with incurable cancer
**Quist, 2015, Denmark**	Prospective study with 1-group design	Health-Related Quality of Life; anxiety; depression	Recruitment by referral from the Department of Oncology, Rigshospitalet, University of Copenhagen	Advanced-stage lung cancer; chemotherapy	114	Median age: 66 (31-88)	50.0	NR	*FACT-L* 94.4 (18.9) EW 15.2 (5.0) *HADS-A* 7.2 (4.4) *HADS-D* 5.3 (3.8)	Patients with advanced-stage lung cancer; patients with inoperable lung cancer
**Jehn, 2012, Germany**	Cross-sectional study	Anxiety; depression	Recruitment not further specified	Metastatic breast cancer; chemotherapy	70	59.9 (SD=10.2)	100	NR	*HADS*	Patients with metastatic breast cancer

Table S3. Data-extraction table of qualitative studies.

Author	Objectives	Study design	Recruitment	Patients	Sample size (n)	Mean age (years)	Female sex (%)	Time since diagnosis	Data analysis	Terminology to refer to patient group
**Brown, 2015*, The Netherlands**	To focus on ongoing, embodied and negotiated processes of sense-making and expectation-construction amidst uncertainty; explore hope, trust and their influence on the illness, medicines and trial experiences of participants	Interviews, field notes	Purposeful recruitment; across phase II or III clinical trials, through a regional hospital and an urban academic hospital	Pancreatic cancer (53.8%), multiple myeloma (23.1%), renal cancer (15.4%), intestinal cancer (7.7%); clinical trial medication (not further specified)	13	63.0	30.8	NR	Interpretative phenomenological approach (IPA)	Advanced-stage cancer diagnoses and limited prognoses
**Catt, 2019*, UK**	To explore experiences of treatment decisions, information provision, perceived benefits and harms of treatment and the effects of these on patients' lives	Interviews	Consecutive sampling; across 20 hospitals in England, Scotland, Wales	Metastatic castrate-resistant prostate cancer (mCRPC); arbirateron, enzalutamide, docetaxel, radium-233, cabazitaxel, steroid switch + abiraterone, goserelin, docetaxel + AZD5363, or enzalutamide + radium-223	37	Partnered men (89.2%): 70.8 (56-89); single men (10.8%): 80.5 (71-91)	0.0	NR	Thematic analysis	Advanced prostate cancer; progressive advanced prostate cancer; treatment has stopped working; the cancer has spread; aggressive prostate cancer; cancer is metastatic
**Grimsbo, 2011**, Norway**	To explore the use and content of patients' e-mail messages sent to oncology nurses and thus gain a ''snapshot'' of patients' experiences of living with cancer as expressed through these messages	Analysis of 276 messages from patients to oncology nurses	Convenience sampling; recruitment via advertisements in the newspaper, on the Norwegian Cancer Society's Website, and its membership magazine, and through information pamphlets mailed to patients from the Norwegian National Cancer Registry	Breast cancer (63.3%) or prostate cancer (36.7%); currently receiving active treatment, not further specified	60 (16.7% patients with metastases)	Breast cancer: 52 (SD=7.9); prostate cancer: 65 (SD=7.6) (35-77)	Breast cancer: 100; prostate cancer: 0.0	n=25 >1 year; n=34 <= 1 year	Qualitative content analysis	Patients with metastases
**Grunfeld, 2012, UK**	To explore the experience and impact of andropause symptoms (particularly hot flashes) among men being treated with ADT	Interviews	Sampling from a clinic database at a large London teaching hospital	Metastatic prostate cancer; receiving androgen deprivation therapy (ADT)	21	78.0 (68-92)	0.0	NR	Qualitative methodology based on the framework approach	Metastatic prostate cancer patients

	for metastatic prostate cancer									
**Holmstrom, 2019*, USA**	To develop a conceptual model that describes patients' experiences of living with chemotherapy-naive metastatic castration-resistant prostate cancer	Interviews	Patients were identified from users of the Health Unlocked social network in USE. Physicians were identified via an external database	Chemotherapy-naive metastatic castration-resistant prostate cancer; hormonal therapy (n=18); anti-androgens (n=13); abiraterone (n=6); bicalutamide (n=7); bisphosphonates (n=11)	19	Median age: 67.0 (51-78)	0.0	Median TSD of metastasis: 4.8 years (range: 1.0-13.0)	Grounded theory	Chemotherapy-naïve metastatic castration-resistant prostate cancer
**Hulin, 2017*, UK, France, Germany, Italy, Spain**	To expand the current knowledge on how relapse affects both patients and physicians	Interviews (including verbal rating scales)	Haematologists were asked to identify and nominate patients with RRMM who would be willing to participate, by using their own judgement	Relapsed or refractory multiple myeloma; targeted therapy: currently receiving lenalidomide (32%) or bortezomib (16%), previously receiving lenalidomide (44%), bortezomib (44%), thalidomide (44%)	50	Median age: 71.0 (51-85)	44.0	NR	Not specified	Patients with relapsed and/or refractory metastatic myeloma (RRMM)
**Kaufman, 2018, Germany, USA**	To provide a comprehensive overview of the impact of MCC and its management on patients' lives prior to diagnosis, at diagnosis, and after diagnosis, and to develop a disease model of adults with metastatic MCC	Interviews	Patients participating in the single-arm, open-label, multicentre, international phase 2 JAVELIN Merkel 200 trial were invited to participate in optional qualitative interviews in all countries (Australia, Austria, Canada, France, Germany, Italy, Spain, Switzerland, and the USA), except Japan where interviews were viewed as culturally insensitive	Stage IV, chemotherapy-refractory, histologically confirmed merkel cell carcinoma; avelumab followed by surgery/radiotherapy	19	72.2 (SD=8.2)	21.0	Mean TSD: 2.3 years (SD: 0.8)	Thematic analysis	Patients with advanced and metastatic MCC; late-stage (advanced or metastatic), non-resectable MCC
**Kvale, 2018, Norway**	To gain insight into the illness narratives of cancer patients, from the day they suspected that something was wrong up to the present day where they are living with incurable cancer,	Interviews	Purposive, non-probability sampling via an outpatient clinic	Metastasized cancers in the palliative phase: colon cancer (n=6), breast cancer (n=4), brain tumor (n=1), prostate cancer (n=1), peritoneum cancer(n=1); palliative,	13	47-79	53.8	0.5-5 years	Thematic narrative analysis, supplied with some elements from structural	Living with life-prolonging chemotherapy; incurable cancer; patients living with life-prolonging chemotherapy; seriously ill cancer patients; non-curable cancer

	undergoing life prolonging chemotherapy			life-prolonging chemotherapy					narrative analysis	
**Levy, 2019, Australia**	To explore the lived experience of being on pembrolizumab treatment for advanced melanoma	Interviews	Purposive sampling; recruitment of patients was across two cohorts (details reported elsewhere)	Metastatic melanoma; pembrolizumab	26	66.0 (41-84)	25.0	NR	Thematic analysis using interpretative phenomenological analysis (IPA)	Metastatic melanoma patients; patients living with advanced melanoma
**Macdonald, 2012*, Canada, USA, Brazil, France, Germany, Russia, Spain**	To explore the experiences and emotions of patients through GIST diagnosis, treatment initiation, disease control, and in some patients, loss of response and therapy switch	Interviews	Recruitment via individual clinical practice centres	Metastatic or unresectable gastrointestinal stromal tumors (GIST); Imatinib	50	NR	NR	NR	Ethnographic approach: multiple, in-context techniques, including individual and group interviews, life histories, and participant observation, were used	Patients with resected or metastatic/unresectable GIST
**Milne, 2019*, Australia**	To understand the experiences of patients with advanced melanoma who received immunotherapy and their carers; and to explore the impact of immunotherapy treatment (in particular side-effects) on patients' and carers' quality of life	Interviews	Purposive sampling from a specialist cancer centre in Melbourne, Australia	Stage IV melanoma; pembrolizumab (57%); nivolumab (13%); ipilimumab (9%); currently not receiving treatment (22%)	23	59.0 (SD=15, 32-86)	22.0	NR	Interpretive description	Patients with advanced melanoma; patients with stage IV melanoma; diagnosis of an incurable/chronic disease
**Mohammed, 2020*, Canada**	To examine how people with late-stage cancer and their healthcare providers enacted the process of medicalisation through engaging in the search for oncological treatments, such as experimental drug trials, despite the incurability of their disease	Interviews, documents, field observations	Purposeful sampling; recruitment from an ambulatory palliative cancer clinic in a comprehensive cancer centre; and snowball sampling for participants with cancer to refer family members, informal caregivers and healthcare providers that they identified as important to their search for treatments	Advanced cancer: prostate cancer, bone marrow cancer, breast cancer, pancreas cancer; receiving a variety of allopathic therapies and informal therapies (not further specified)	7	Late 30s-early 70s	43.0	6 months - 5 years	Inductive coding, guided by a critical discourse analytical framework	Advanced cancer, incurable illness, people with late-stage cancer, terminal cancer, chronic illness that one could potentially survive, people with advanced, metastatic and life-limiting cancer

**Mosher, 2013, USA**	To identify concerns among distressed women with metastatic breast cancer with an emphasis on their experience of physical and emotional distress, social constraints, and existential issues	Analysis of 176 essays from home-based expressive writing trial	Recruitment via an invitational letter for an expressive writing intervention trial, after giving approval for contact via their oncologist	Stage IV breast cancer; hormonal therapy (77.3%), chemotherapy (97.7%)	44	57.9 (SD=12.1)	100	Mean TSD: 4.1 (SD: 3.1)	Theoretical thematic analysis of essays	Living with metastatic breast cancer; incurable illness; long-term survivor; advanced cancer patients
**Pujol, 2018, France**	To investigate patient's thoughts and attitudes that determine the decision to undergo second-line chemotherapy	Interviews	Purposive sampling; consecutive participants admitted to receive chemotherapy (second-line or third-line/palliative intent) for a lung cancer in the thoracic unit of the Montpellier academic hospital	Advanced non-small cell or small cell lung cancer; second-line or palliative chemotherapy	33	61.8 (SD=7.9)	33.0	NR	Grounded theory	Cancer patients in a palliative situation
**Finlayson, 2017, USA**	To understand the lived experience of being aware of disease status among women with recurrent ovarian cancer	Interviews	Purposive sampling; recruitment from the outpatient gynecological medical service of one large metropolitan cancer center via six different medical oncologists	Recurrent ovarian cancer; currently receiving chemotherapy	12	58.0 (44-74)	100	< 2 years: 8.3%; 2-3 years: 33.3%; 3-4 years: 16.7%; 4-5 years: 8.3%; 5+ years: 33.3%	A modified Colaizzi's seven-step method	Advanced(-stage)ovarian cancer patients; women with recurrent ovarian cancer
**Thorne, 2013, Canada**	To glean insights from the patient perspective as to what constitutes helpful and unhelpful clinical communication, and to determine how patients' needs and preferences in relation to such communication change over time within the cancer journey	Interviews	Purposive sampling; voluntary samples and recruited through multiple approaches	Chronic advanced metastatic cancer: breast cancer (n=8); hematological (n=5); gastrointestinal (n=2); receiving novel cancer therapies (not further specified)	15	55.5 (32-72)	NR	NR	Interpretive description	Persons surviving for extended periods with advanced disease; incurable and life-limiting metastatic conditions; patients living longer with serious disease; chronic advanced cancer; chronic metastatic cancer; longstanding advanced metastatic cancer patients
**Wickersham, 2014, USA**	To explore the process of medication-taking for adults with non-small cell lung cancer receiving oral EGFRI	Interviews	Purposive sampling; recruitment at two outpatient lung cancer clinics at the National Cancer Institute	Non-small cell lung cancer; EGFRI-treatment erlotinib	13	70.5 (52-83)	61.5	NR	Constant comparative analysis, grounded theory,	Surviving with lung cancer; surviving with metastatic cancer; life-limiting illness without cure; not survive after,

	therapy, to develop a grounded theory that described and explained the process of medication-taking in this patient population		designated cancer center						questioning the data, dimensional analysis, matrix construction, writing case titles and story summaries, and a literature review	but with lung cancer; chronic life-threatening condition
**Wong, 2019, Australia**	To explore real-world experiences of patients with advanced melanoma currently receiving pembrolizumab, focussing on factors that influenced their treatment decision-making	Interviews	Purposive sampling; recruitment of patients from a specialist cancer center in Melbourne, Australia	Stage IV melanoma; pembrolizumab; prior treatment ipilimumab (22%) or dabrafenib/trametinib (9%)	23	68.0 (SD=12, 34-92)	13.0	NR	Thematical analysis	Stage IV melanoma; advanced melanoma
**Al Achkar, 2020, USA**	To characterise the unmet needs of patients with advanced lung cancer on targeted therapy and to explore how their healthcare experiences with clinicians and care teams can be improved	Interviews	Recruitment via online closed oncogene-focused lung cancer groups of patients and their caregivers (Facebook, ROSOneder, EGFR Resisters)	Advanced or metastatic non-small cell lung cancer stage IV (95%) or stage IIIb (5%); targeted therapy (not further specified)	39	Median age: 48 (30-75)	71.8	Median TSD: 21 months (3-81)	Critical theory-based analysis methods	Patients with advanced lung cancer on targeted therapies
**Gray, 2020, South Africa**	To report experiences of men in South Africa treated with ADT to share their perceived effects of the treatment	Interviews	Recruitment via an urban oncology clinic in Pretoria, South Africa	Advanced prostate cancer; androgen deprivation therapy (ADT)	22	63-78	0.0	NR	NR	Prostate cancer patients
**Poort, 2021, USA**	To explore patients' experiences of PARPi-related fatigue and its impact on their lives	Interviews	Purposive sampling; potentially eligible participants were identified using an EPIC workbench that identified patients with a PARPi listed in the electronic records' medication section	Advanced ovarian cancer stage I-II (22%), stage III (70%), stage IV (13%); receiving oral PARP inhibitors	23	63.0 (50-86)	100	Mean TSD: 3 years (0-11)	Thematic analysis	Ovarian cancer patients on maintenance PARPi
**Walsh, 2021, USA**	To explore how lung cancers survivors engage in online support	Interviews	Purposive sampling; recruitment via oncogene-specific	Advanced lung cancer stage IV (95%) or stage IIIb (5%); targeted	40	Median age: 49.0 (30-75)	70.0	Median TSD: 19.5	Thematic analysis	Lung cancer survivors

	communities and what the psychological, social and physical impacts of such engagement are		online lung cancer support groups: EGFR Resisters, ALK-Positive Facebook Group and the ROS1ders	therapies (not further specified)				months (3-152)		
**Buiting, 2013, The Netherlands**	To explore the extent to which patients have a directing role in decisions about chemotherapy in the palliative phase of cancer and (want to) anticipate on the last stage of life	Interviews	Purposive sampling; via a palliative care nurse who traced and invited potentially eligible patients	Colon cancer (66.6%) or breast cancer (33.3%); indication second line chemotherapy (n=1); second line chemotherapy (n=4); indication third line chemotherapy (n=1); indication third line immunotherapy (n=1); third line chemotherapy (n=2); (indication) third/fourth line chemo-immuno-/targeted therapy (n=2)	15	65.0 (48-85)	53.3	NR	Thematic content analysis	Patients with advanced colorectal and breast cancer
**Karlsson, 2014, Sweden**	To interpret meanings of existential uncertainty and certainty for people diagnosed with advanced gastrointestinal cancer and receiving palliative treatment	Interviews	Recruitment via an oncological outpatient clinic that specialises in palliative care for gastrointestinal cancer	Gastrointestinal cancer with various metastases; palliative treatments (not further specified)	14	49-79	50.0	2 months - 20 years	Naive reading, structural analysis and interpreted whole (in line with Ricoeur's theory of interpretation)	People diagnosed with advanced gastrointestinal cancer and receiving palliative treatment
**Svensson, 2009, Sweden**	To explore psychological reactions and coping at disease progression after first-line chemotherapy among women with metastatic breast cancer participating in a randomized clinical trial	Interviews	Women included in a randomized phase III study were asked to participate	Metastatic breast cancer; chemotherapy: epirubicin and paclitaxel with or without capecitabine	20	56.3 (6.2)	100	TSD to PD first line: 2 - 20 years	Content analysis	Women with metastatic breast cancer
**Brown, 2015*, The Netherlands**	To focus on ongoing, embodied and negotiated processes of sense-making and expectation-construction amidst uncertainty; explore hope, trust and their influence on the	Interviews, field notes	Purposeful recruitment; across phase II or III clinical trials, through a regional hospital and an urban academic hospital	Pancreatic cancer (53.8%), multiple myeloma (23.1%), renal cancer (15.4%), intestinal cancer (7.7%); clinical trial medication (not further specified)	13	63.0	30.8	NR	Interpretative phenomenological approach (IPA)	Advanced-stage cancer diagnoses and limited prognoses

	illness, medicines and trial experiences of participants									
**Catt, 2019*, UK**	To explore experiences of treatment decisions, information provision, perceived benefits and harms of treatment and the effects of these on patients' lives	Interviews	Consecutive sampling; across 20 hospitals in England, Scotland, Wales	Metastatic castrate-resistant prostate cancer (mCRPC); arbirateron, enzalutamide, docetaxel, radium-233, cabazitaxel, steroid switch + abiraterone, goserelin, docetaxel + AZD5363, or enzalutamide + radium-223	37	Partnered men (89.2%): 70.8 (56-89); single men (10.8%): 80.5 (71-91)	0.0	NR	Thematic analysis	Advanced prostate cancer; progressive advanced prostate cancer; treatment has stopped working; the cancer has spread; aggressive prostate cancer; cancer is metastatic
Grimsbo, 2011**, Norway	To explore the use and content of patients' e-mail messages sent to oncology nurses and thus gain a ''snapshot'' of patients' experiences of living with cancer as expressed through these messages	Analysis of 276 messages from patients to oncology nurses	Convenience sampling; recruitment via advertisements in the newspaper, on the Norwegian Cancer Society's Website, and its membership magazine, and through information pamphlets mailed to patients from the Norwegian National Cancer Registry	Breast cancer (63.3%) or prostate cancer (36.7%); currently receiving active treatment, not further specified	60 (16.7% patients with metastases)	Breast cancer: 52 (SD=7.9); prostate cancer: 65 (SD=7.6) (35-77)	Breast cancer: 100; prostate cancer: 0.0	n=25 >1 year; n=34 <= 1 year	Qualitative content analysis	Patients with metastases

Table S4. Data-extraction table of mixed-methods studies.

Author	Study design	Psychosocial outcomes	Recruitment	Patients	Sample size	Mean age (years)	Female sex (%)	Time since diagnosis	Quantitative baseline results (mean (SD))	Terminology patient group
**Ito, 2018, UK, France, Germany, Italy, Spain**	Cross-sectional mixed-methods: interviews, questionnaires	Quality of Life; Experiences of chemotherapy in combination with androgen deprivation therapy	Recruitment via five European countries	Metastatic hormone-sensitive prostate cancer; chemotherapy (docetaxel) in combination with androgen deprivation therapy	Interviews: 31; questionnaires: 161	Interviews: 41-50 (n=1), 51-60 (n=11), 61-70 (n=11), 71-80 (n=8); questionnaires: ≤40 (n=6) 41-50 (n=8), 51-60 (n=30), 61-70 (n=64), 71-80 (n=50), ≥ 81 (n=3)	0.0	NR	*FACT-P* 92.4 (21.9) EW 15.0 (5.7) *EQ-5D-5LVAS* 60.7 (17.3)	Men with metastatic hormone-sensitive prostate cancer; metastatic hormone-sensitive prostate cancer patients
**Paterson, 2017, Scotland**	Cross-sectional mixed-methods: interviews, questionnaires	Quality of Life; Experience of unmet supportive care needs	Purposive sampling; recruitment via a main cancer centre in Scotland	Locally advanced and metastatic prostate cancer; hormonal treatment	Interviews: 8, questionnaires: 31	80.1 (SD= 6.9)	0.0	1-2 years	*EORTC-QLQ-C30* GH 69.6 (23.9) EF 88.2 (16.9)	Men affected by prostate cancer on hormone therapy
**Ihrig, 2020, Germany**	Cross-sectional mixed-methods: interviews, questionnaires	Possible prejudices, biases, unrealistic expectations and misconceptions about immunotherapy	Consecutive sampling; recruitment via the university hospital Heidelberg	Advanced cancer: immune checkpoint inhibitors (ICI) (n=53): dermatological tumours (43%), thoracic tumours (38%), gastrointestinal tumours (4%), other tumours (15%), metastasis (96%); chemotherapy (CT) (n=55): thoracic tumours (38%), gastrointestinal tumours (49%), other tumours (13%). metastasis (85%)	161	59.5 (S=11.7) versus 62.4 (SD=11.0) for ICI versus CT	40.0 for both ICI and CT group	NR	No outcomes of validated measures were reported	Advanced-stage cancer patients

**Komatsu, 2020, Japan**	Longitudinal mixed-methods RCT: focus group interviews, questionnaires	Self-efficacy; Quality of Life; psychological distress	Recruitment via outpatient lists by primary physicians, at the outpatient clinics of three cancer centres and one university hospital in Japan	Metastatic breast cancer; chemotherapy or targeted therapy	155	57.0 (SD= 12.2) versus 59.4 (SD= 11.6) for the nurse-led medication self-management programme (n=78) versus control group (n=76)	100	NR	*GSE* 26.7 (0.6) versus 25.7(0.8) *FACT-B* 90.3 (2.4) versus 92.6 (2.1) *6-item Kessler 6 (K6)* 5.0 (0.5) versus 5.3 (0.5) for the nurse-led medication self-management programme versus control group	Patients with metastatic breast cancer (undergoing oral anticancer treatment)
**Mieras, 2020, The Netherlands**	Cross-sectional mixed-methods: interviews, questionnaires	Quality of Life	Purposive sampling for the interviews; recruitment via clinic schedules by treating oncologists via one academic and five non-academic hospitals across the Netherlands	Metastatic lung cancer; chemotherapy (36%), immunotherapy (47%), TKIs (17%)	266	65.0 (SD=9.0)	45.0	NR	*EORTC-QLQ-C30* baseline means NR	Patients with metastasized lung cancer; patients diagnosed with metastatic lung cancer
**Charalambous, 2016, Cyprus**	Cross-sectional mixed-methods: interviews, questionnaires	Quality of Life	Recruitment via the Out-Patients Oncology Clinics of two public hospitals in Cyprus; randomly selected patients (from the quantitative	Advanced prostate cancer; chemotherapy with docetaxel as primary or combination chemotherapy	Interviews: 15; questionnaires: 148	40–50 (16.9%); 51–60 (25.0%); 61–70 (31.8%); >70 (26.4%)	0.0	6 months-3 years: 59.5%; 4-6 years: 30.4%; 7-10 years: 4.1%; >10 years: 6.1%	*EORTC-QLQ-C30* GH 56.7 (19.0) EF 48.25 (26.55)	Patients diagnosed with advanced prostate cancer (during the period of active treatment with chemotherapy)

			sample) were interviewed on the basis of the findings from the statistical analysis of the quantitative data							
