# Supplementary figures and images for: Psychosocial Aspects of Living Long Term with Advanced Cancer and Ongoing Systemic Treatment: A Scoping Review
Source: Cancers (Basel). 2022 Aug 11;14(16):3889. doi: 10.3390/cancers14163889 (PMC9405683; doi:10.3390/cancers14163889)

**Figure S1.** PRISMA-ScR Flow chart.

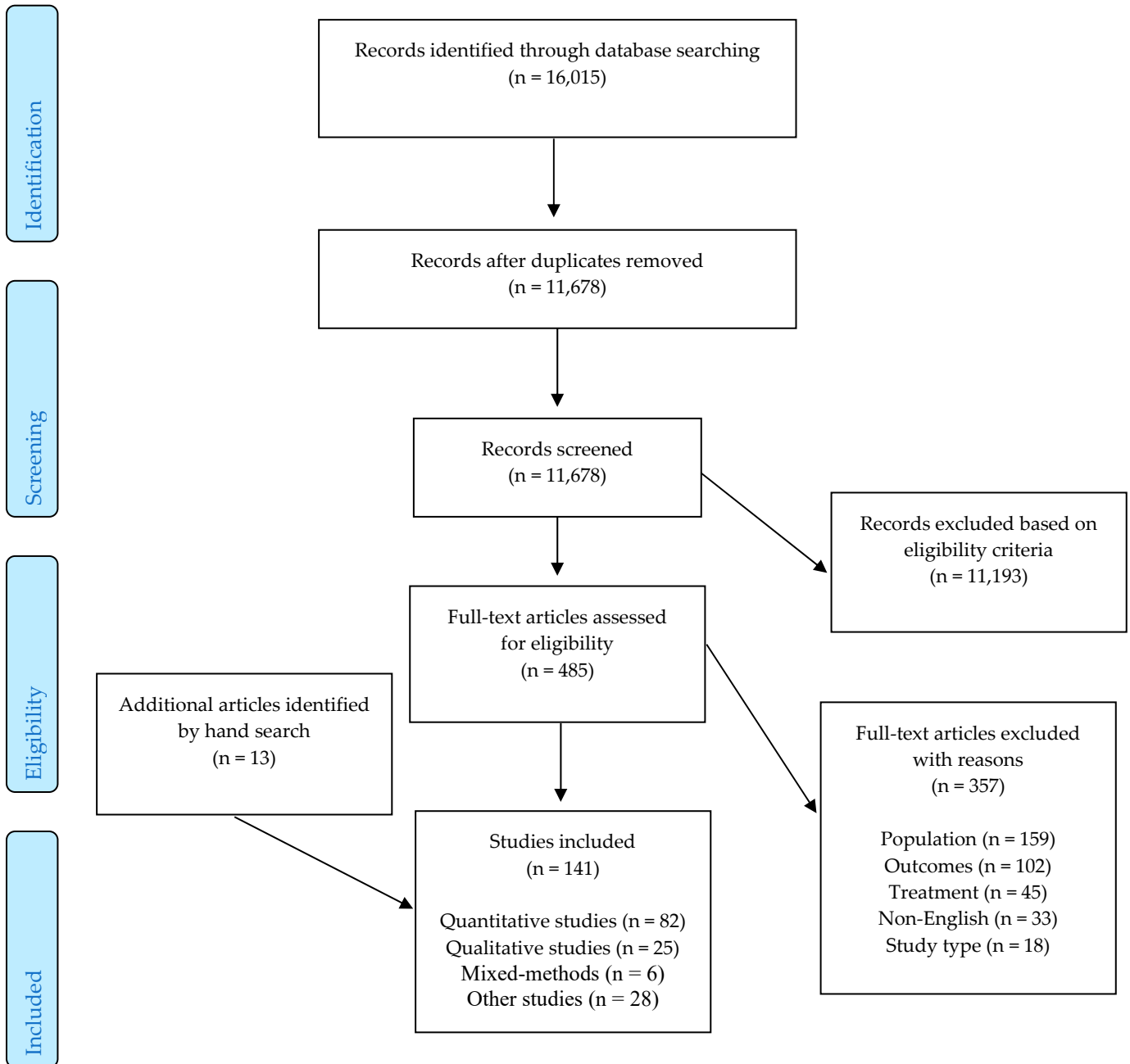

Supplement: Supplementary file 1 [file cancers-14-03889-s001.zip › cancers-1825415-supplementary/Supplementary File S2. PRISMA-ScR flow chart.pdf]
